# Renal transplantation in Birt-Hogg-Dubé syndrome: should we?

**DOI:** 10.1186/s12882-018-1064-5

**Published:** 2018-10-16

**Authors:** Joana Coutinho, Joaquim de Sa, Filipe Castro Teixeira, Catarina Reis Santos, Raquel Sa Chorão, Rui Alves Filipe, Ernesto Fernandes Rocha

**Affiliations:** 1 Hospital Amato Lusitano, Rua Professora Maria Amalia Fevereiro Lote A 105, 4°direito, 6000-472 Castelo Branco, Portugal; 2 Centro Hospitalar Universitario de Coimbra, Coimbra, Portugal

**Keywords:** Dialysis and transplantation, Genetics, Cancer

## Abstract

**Background:**

Birt-Hogg-Dubé (BHD) Syndrome is a rare genodermatosis caused by a mutation on folliculin gene, with a strong link to renal cancer. To date few patients with such condition have reached dialysis stage, as nephron-sparing surgery is usually possible at the time of diagnosis. To our best knowledge no patient with BHD syndrome has been submitted to renal transplantation.

**Case presentation:**

We report the case of a woman diagnosed with multifocal bilateral renal cell carcinoma that underwent bilateral radical nephrectomy and was started on a regular hemodialysis program at the age of 29. While on hemodialysis program she was diagnosed clinically with BHD syndrome and molecular testing confirmed an heterozygous mutation on FLCN gene. The patient has been kept on surveillance program for 2 years with no clinical complications from the genetic syndrome and in complete remission from renal cancer. Though there has not been any report of a patient with BHD being transplanted, risks and benefits for this patient were weighted. She has been considered apt by the transplant team and is currently waitlisted for cadaveric renal transplantation.

**Discussion:**

It is a matter of discussion which should be cancer-free period for anephric patients with an inherited cancer syndrome to be candidates for renal transplant. So far BHD syndrome has not been causally associated with any other neoplastic disorder elsewhere. Accepting cancer biology is very complex and knowledge of the behaviour of this genetic syndrome is limited to a few cases reported worldwide, the authors believe that renal transplantation is the best treatment option for this young patient. The choice of post transplantation immunosuppression is debatable, but considering experience in other inherited cancer syndromes a maintenance scheme with mTOR inhibitor will be favoured.

## Background

Young patients with multifocal, bilateral kidney cancers should be screened for inherited renal cancer syndromes. Although the management of the tumour invariably involves nephrectomy (nephron-sparing or not) with or without adjuvant therapies, the patient prognosis is greatly influenced by the inherited syndrome [1–3]. Hereditary kidney cancer syndromes follow different clinical courses and respond differently to therapy compared to sporadic kidney cancers. Most importantly, the molecular diagnosis may reveal other genetic underpinnings of the disease and its phenotypic variations alerting for possible clinical consequences. Molecular identification of involved genes will be determinant for prediction of future clinical implications, risk of recurrence and evaluation of patient’s candidacy to renal transplant.

## Case report

We report the case of a 29-year-old female with a past medical history of smoking (5-pack-years) and a spontaneous pneumothorax four years earlier. The aforementioned pneumothorax was complicated by continuous re-expansion of the air chamber, resolving only after atypical basal segment lung resection and pleurodesis by video-assisted thoracotomy. The patient had no family history of spontaneous pneumothorax, cancer or consanguinity.

On April of 2014 on a routine gynaecological consultation a pelvic ultrasonography revealed multiple and bilateral hypoechogenic renal masses. The patient was immediately referred to urology care. The patient underwent percutaneous ultrasound-guided kidney biopsy. Histology report identified cells suggestive of carcinoma. On computer tomography multiple masses were present on both kidneys and bilateral nephrectomy was scheduled. Two days after surgery the patient was started on a regular haemodialysis program.

The pathology report of nephrectomised samples revealed the right kidney had nine intra-renal tumoural masses, not invading the capsule and corresponding to renal cell carcinoma of chromophobe cell type. The left kidney had nineteen intra-renal masses with the same histology, without capsular involvement. The tumour was staged according to TMN classification system, 7th edition, as T1b Nx Mx, with no residual tumour. Since surgical margins were negative and there were no signs of distant metastatic disease, the patient was not proposed for adjuvant chemotherapy.

**Fig. 1:**
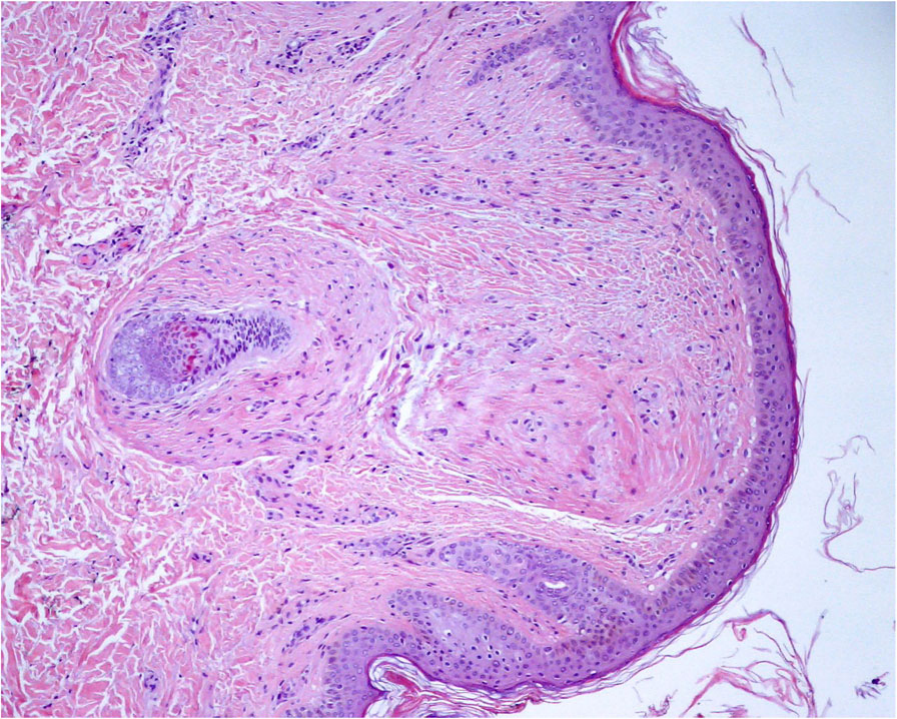
10× Amplification of Trichodiscoma, biopsied from the patient cervical region

While on regular haemodialysis, multiple small whitish papules were noticed predominantly on the head and neck. These papules had been slowly appearing after the age of 20. Skin biopsy confirmed hair follicle benign tumours of trichodiscoma type [Fig. 1].

Considering the patient past medical history and the skin lesions, Birt-Hogg-Dubé (BHD) syndrome was considered. The hallmark features of this genodermatosis are benign skin tumours of fibrofolliculoma, trichodiscoma or acrochordon type [1, 2, 4]. This syndrome is also characterized by a strong link to renal cancer and spontaneous pneumothoraxes, with some authors reporting a 7-fold increased risk for developing renal tumours and 50-fold for developing spontaneous pneumothoraxes compared to normal population [5]. The renal tumours are multifocal or bilateral in the majority of patients with BHD and the most typical histological types are pure chromophobe, oncocytomas and hybrid chromophobe renal cell carcinoma [1, 3, 6]. According to the European Consortium guidelines, this patient had 1 major criteria and 2 minor criteria for BHD syndrome [7].

Genetic analysis confirmed an heterozygote mutation on FLCN gene, in exon 6 (C.573_574delinsT). The family was screened for the mutation and it was found on 3 other family members (father, sister, first degree cousin), which are now on surveillance program.

The patient has been on dialysis for 2 years now. Though there has not been any report of a patient with BHD being transplanted, risks and benefits for this patient were weighted. She has been considered apt by the transplant team and is currently waitlisted for cadaveric renal transplantation.

## Discussion

Definite diagnosis of Birt-Hogg-Dubé Syndrome requires genetic testing for folliculin mutations. Genetic transmission of this syndrome occurs through autosomal dominant pattern. To date, 194 unique DNA variants of the folliculin gene (FLCN) have been reported as responsible for this syndrome [2]. The FLCN mutation detection rate is 88% [8], due to a great variation in the expression of FLCN gene both within and between families with BHD [7].

Though it had been hypothesized FLCN protein had tumour suppressor properties and could be one of the major proteins regulating the mTOR pathway, only recently was FLCN established as a tumour suppressor gene that fits the classic two-hit model [9]. In addition to FLCN germline mutation, a second somatic mutation in the remaining FLCN allele has been found in tumour cells [10, 11]. FLCN interacts mainly with 3 proteins: folliculin interacting proteins (FNIP1 and FNIP2) and 5’-AMP activated protein kinase. Studies in vitro suggest FLCN has a role facilitating mTOR activation at the lysosome surface, however further studies are needed to clarify mechanisms involved in mTOR signalling by the FLCN/FNIP complex and possibly validate the role of mTOR inhibition in tumour progression suppression [12, 13].

Family screening for carriers of FLCN mutations is of outmost importance since kidney cancer is the most lethal potential complication of this syndrome [5, 7]. Nephron-sparing surgery should be favoured when possible [14, 15]. Surveillance is recommended to start after the age of 20, although the methods and optimal programme for surveillance are not yet established [7]. As for the respiratory complications, the role of tobacco on increasing the risk of pneumothorax on BHD-carriers is yet not established, however lifetime smoking eviction is strongly encouraged for all BHD-carriers [7]. BHD-carriers who already had a spontaneous pneumothorax are recommended to avoid exposure to great atmospheric pressure gradients, as in deep-sea diving. Currently there is no evidence against occasional air travelling.

Folliculin protein is expressed in a variety of tissues contrary to what was previously believed, however it is only known to have pathological significance in renal, lung and skin tissue [16]. BHD-associated renal cancer shares some of the clinical features of the tuberous sclerosis complex syndrome, which on its own also regulate proteins involved in the mTOR pathway, and should be part of the differential diagnosis when considering BHD.

To our knowledge there hasn’t been any report of a BHD patient having a kidney transplant, however there is some evidence of successful renal transplantation in patients with other inherited kidney cancer syndromes such as Von Hippel Lindau and Tuberous sclerosis syndrome [17–19]. The main concern regarding transplantation in these patients is malignancy recurrence or de novo emergence following post-transplantation immunosuppression. Though BHD syndrome has not been causally associated with cancer elsewhere apart from the kidney, the effects of immunosuppression on this complex and not completely elucidated cancer syndrome are unknown.

Choice of post-transplant immunosuppressive therapy should be individualized considering the patient malignancy risk. Calcineurin inhibitors have been associated with dose-dependent increased risk of malignancy and mTOR inhibitors have been proven to have a less cancerogenic profile and even anti-tumour properties, being used to delay progression of metastatic renal cell cancer [20]. There is evidence that mTOR inhibitors should be the favored immunosuppressant in patients with loss of VHL function [20] and has been used in transplantation of patients with Von Hippel Lindau disease.

## Conclusions

It is a matter of discussion which should be cancer-free period for anephric patients with an inherited cancer syndrome to be candidates for renal transplant. KHA-CARI recommends 5 years, other authors recommending 2 years [21, 22]. As BHD syndrome has not been causally associated with any other neoplastic disorder elsewhere [1, 7, 8], the authors defend that no cancer-free period should be needed, as long as the patient is completely anephric. Accepting cancer biology is complex and knowledge of the behaviour of this genetic syndrome is limited to a few cases reported worldwide, the authors believe that renal transplantation is the best treatment option for this young patient and probably will opt for a post-transplant immunosuppressive maintenance scheme with a mTOR inhibitor.

## Abbreviations

BHD: Birt-Hogg-Dubé
FLCN: Folliculin gene
KHA-CARI: Kidney Health Australia – Caring for Australasians with Renal Impairment
mTOR: Mechanistic target of rapamycin
VHL: Von Hippel Lindau
